# Social dominance modulates eavesdropping in zebrafish

**DOI:** 10.1098/rsos.150220

**Published:** 2015-08-26

**Authors:** Rodrigo Abril-de-Abreu, Ana S. Cruz, Rui F. Oliveira

**Affiliations:** 1 Instituto Gulbenkian de Ciência, Rua da Quinta Grande 6, Oeiras 2780-156, Portugal; 2 ISPA—Instituto Universitário, Rua Jardim do Tabaco 34, Lisboa 1149-041, Portugal; 3 Champalimaud Neuroscience Programme, Avenida de Brasilia, Lisboa 1400-038, Portugal

**Keywords:** social eavesdropping, dominance, social learning, individual recognition, attention, zebrafish

## Abstract

Group living animals may eavesdrop on signalling interactions between conspecifics and integrate it with their own past social experience in order to optimize the use of relevant information from others. However, little is known about this interplay between public (eavesdropped) and private social information. To investigate it, we first manipulated the dominance status of bystander zebrafish. Next, we either allowed or prevented bystanders from observing a fight. Finally, we assessed their behaviour towards the winners and losers of the interaction, using a custom-made video-tracking system and directional analysis. We found that only dominant bystanders who had seen the fight revealed a significant increase in directional focus (a measure of attention) towards the losers of the fights. Furthermore, our results indicate that information about the fighters' acquired status was collected from the signalling interaction itself and not from post-interaction status cues, which implies the existence of individual recognition in zebrafish. Thus, we show for the first time that zebrafish, a highly social model organism, eavesdrop on conspecific agonistic interactions and that this process is modulated by the eavesdroppers' dominance status. We suggest that this type of integration of public and private information may be ubiquitous in social learning processes.

## 1. Introduction

Public information is widely available at low cost to animals living in social groups [1], where most social signalling events are within the range of other members [2]. For instance, bystanders can potentially extract information from signalling interactions between conspecifics and use it to adjust their subsequent behaviour towards the observed individuals, without the costs of first-hand experience. This ability to collect and use adaptively relevant information from others' interactions (social eavesdropping) is hence expected to impact the Darwinian fitness of the animal.

Social eavesdropping has been investigated in different species, mostly in the contexts of mate choice and male–male territorial interactions [3]. The use of agonistic interactions for the study of social eavesdropping provides several advantages, since they are relevant for the establishment of dominance hierarchies that regulate the access to resources such as reproduction sites, mates or food. Furthermore, agonistic interactions are a salient social event, easy to manipulate experimentally and where the emergence of winners and losers provides an honest signal of competitive ability. This gives eavesdroppers the opportunity to assess the relative fighting ability of potential rivals, without directly engaging in a fight themselves [4].

Moreover, one might expect that integration of eavesdropped information with information gathered by direct past experience with others will enable a better adaptive response to the social environment. However, little is known about this interplay between public and private social information [5]. We investigated this question by assessing the occurrence of social eavesdropping on agonistic interactions of zebrafish and its potential modulation by the eavesdroppers' own dominance status.

This species is a highly social model organism [6], which lives in groups with structured dominance hierarchies and is an emerging experimental model in social neuroscience and neuroethology [7]. Previous work in our laboratory showed that zebrafish are tuned to be attentive to conspecifics' fighting interactions and are attracted by specific movement or form features present in those interactions [8]. Namely, we have shown that the fighters' structure of movement during the assessment phase of the fight elicits a higher response from bystanders than the post-resolution chasing phase, and that this response also seems to be dependent on the fighters' form features (e.g. fish shape). Furthermore, zebrafish exhibit behavioural flexibility dependent on past social experience, as shown by the existence of winner and loser effects [9]. Based on these results, we developed an eavesdropping paradigm, using attentional measures of directionality and proximity towards the stimulus. We tested if bystander zebrafish, who themselves had lost or won a fight as their latest social experience, would visually extract and differentially use information about the winners and losers of observed fighting interactions.

## 2. Material and methods

### 2.1 Animals and housing

Wild-type (AB) male zebrafish (*Danio rerio*), 9 to 12 months old, bred at Instituto Gulbenkian de Ciência (IGC, Oeiras, Portugal) were used. Fish were kept in mixed sex shoals in environmentally enriched (gravel substrate, artificial plants, rocks and refuges) stock tanks at 25°C, under a 12 L:12 D photoperiod. Fish were fed three times a day, except on the days of the experiments.

### 2.2 Status manipulation set-up

Two fight tanks (15×15 cm), with a 9 cm water depth, were placed inside a bigger tank (50×25 cm) containing a mixed sex shoal of 30 individuals to act as an audience (see electronic supplementary material, figure S1). Each fight tank was divided in half by an opaque removable partition. When lowered, the partition prevented visual and physical contact between two isolated fish but allowed chemical communication. When lifted, the fish could interact and fight. The audience allowed the fighting fish to assess their dominance status in a shoal-like context, similar to their ‘natural’ stock tank environment, while also reducing their stress levels prior to the interaction. A video camera recorded all fights.

### 2.3 Eavesdropping set-up

**Figure 1.**
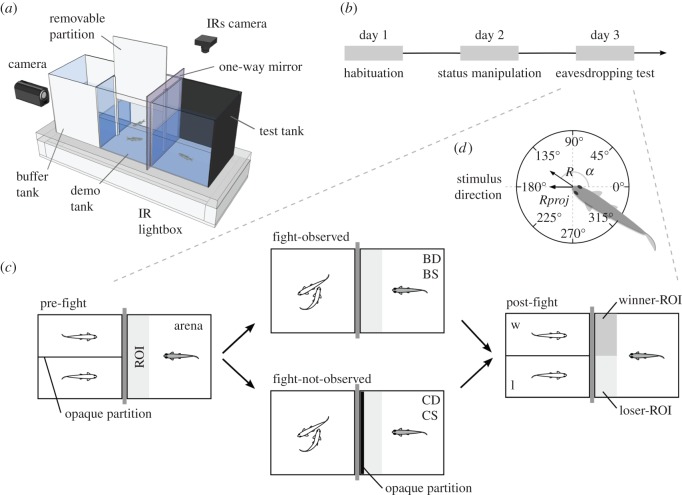
Social eavesdropping paradigm. (*a*) Three-dimensional diagram of the experimental set-up. Left wall coverings of the test and demo tanks are removed for easier visualization. (*b*) Timeline of experimental procedure. (*c*) Schematic of eavesdropping test (day 3), composed of three 30 min stages: pre-fight, fight-observed/fight-not-observed and post-fight. Demonstrator fish in white and focal fish in grey, belonging to four conditions: bystander dominant (BD), bystander subordinate (BS), control dominant (CD) and control subordinate (CS). At the post-fight stage, the side of winner (w) and loser (l) demonstrators is randomized. (*d*) Schematic of a focal fish's mean resultant directional vector, composed of the vector's length *R* and mean angle *α*(0° opposite and 180° directed towards the stimulus), and *R* projected onto 180° (*Rproj*).

A test tank (13×13×17 cm) was placed facing a demonstrator tank (30×15×17 cm), with a one-way mirror in-between (figure 1*a*). This allowed for a bystander focal fish placed in the test tank to see a demonstrator fish pair, without itself being seen. It also prevented interactions between demonstrators and bystanders. No chemical communication was possible as the tanks were self-contained. The demonstrator tank was divided in half by a transparent partition. The outer half (buffer tank) buffered the fish from interference of spurious external cues and minimized stress from the experimenter's manipulations; the half adjacent to the test tank (demo tank) was further divided in two by an opaque removable partition and held the demonstrator fish. A top-view infrared sensitive (IRs) camera recorded the test tank and a front-view camera recorded the demonstrator tank. An IR custom-built lightbox increased contrast between the background of the test tank and the focal fish (when video recording from above) for offline tracking. The complete experimental set-up comprised four adjacent replicas of the described setting, one for each experimental condition.

### 2.4 Experimental procedure

On day 1, two pairs of unfamiliar male zebrafish matched in size were placed in the status manipulation set-up, separated by opaque partitions and left to habituate overnight (figure 1*b*). On day 2, the opaque partitions were lifted and the fish dyads could fight [9]. Once dominance was established (see electronic supplementary material, video S1), they were again separated, placed individually (dominants and subordinates) in the eavesdropping set-up's test tanks (figure 1*a*) and randomly assigned to bystander or control treatments. Therefore, four focal conditions were created: bystander dominant (BD), bystander subordinate (BS), control dominant (CD) and control subordinate (CS). In parallel, four male pairs matched in size were isolated in each demonstrator tank to be used as fighters. Each focal fish could see the corresponding demonstrator pair to allow familiarization. On day 3, the eavesdropping test (figure 1*b*,*c*) started with a 30 min pre-fight stage (baseline), where each focal fish had full view of the separated demonstrators, followed by a 30 min fight-observed stage for the bystander treatment fish and a fight-not-observed stage for the controls, which were prevented from observing the fight by an opaque partition. Afterwards, winner and loser demonstrators were again separated. The fights were video recorded for later determination of the winner's and loser's end position in their tank (left or right). In the post-fight stage, focal fish were allowed to observe for 30 min the separated winners and losers of the corresponding fights. Focal fish were video recorded at all stages and their behaviour was video tracked (see electronic supplementary material, video S2) and analysed. A total of 71 focal fish were analysed (*n*=19 for the BD condition; *n*=17 for BS; *n*=18 for CD; and *n*=17 for CS).

### 2.5 Behavioural analysis

Baseline (pre-fight) and fight observation values of *Rproj* and speed (measure of motor activity) were determined in the total tracked area (arena) together with the time spent in a region of interest (ROI) closest to the demonstrator tank (figure 1*c*). The demonstrators' latency to fight (time to first aggressive display) and fight resolution time (from first display to winner–loser decision) were determined for all dyads. One-way ANOVAs were used to compare all conditions.

Eavesdropping effects were investigated at the post-fight stage by comparing two defined ROIs closest to the winner (winner-ROI) and loser (loser-ROI) demonstrator's sides (figure 1*c*). Directional focus towards each demonstrator (*Rproj*), time spent in each region and mean orientation (*α*) were determined for each focal fish and condition. *Rproj* was defined as the projection of the fish's mean resultant directional vector's length *R* onto the demonstrator tank's direction (180°) and ranged from 1 to −1. Positive values indicate directionality towards the stimulus direction, negative values away from it and null values no directional focus (figure 1*d*; see the electronic supplementary material for details). Mixed-design ANOVAs were used to check effects and interactions of treatment, status and side (within-subjects variable) on the attentional measures. Planned contrasts were used within the model for specific comparisons between the winner-ROI and loser-ROI. Effect sizes were determined by Cohen's *d*.

Trend effects from observing or not observing a fight were analysed by comparing pre-fight with post-fight for each condition. Mixed-design ANOVAs and planned contrasts were used. Pearson correlations were performed between the latencies to fight, resolution times and subsequent levels of the bystander fishes' directional focus towards the losers at the post-fight stage.

Behavioural parameters were represented as mean±s.e.m., except mean angles represented as mean±95% CI, when directionality was significant. Statistical significance was considered for *p*<0.05. All analyses were performed using Matlab R2012b (MathWorks) with the CircStat toolbox [10], Statistica v. 12 (Statsoft, Inc.), SPSS Statistics 22 (IBM) and Oriana 4 (Kovach Computing Services).

See the electronic supplementary material for an extended description of material and methods.

## 3. Results

### 3.1 Bystanders' behaviour before the fights

**Figure 2.**
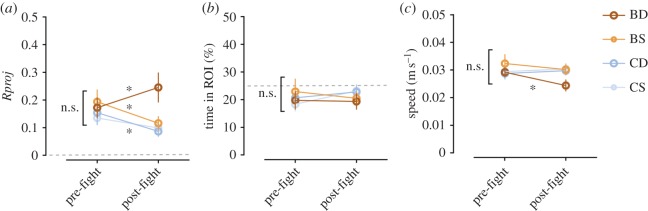
Baseline behavioural results and trend comparisons with post-fight stage. (*a*) *Rproj* in arena. Dashed grey line indicates no directionality. (*b*) Mean time spent in ROI. Dashed grey line indicates chance level (25%). (*c*) Mean speed in the arena. Conditions: BD, BS, CD and CS. Mean±s.e.m. represented. n.s., non-significant, *p<0.05, **p<0.01.

Baseline (pre-fight) analysis of the focal fishes' behaviour in the total arena and ROI did not reveal any differences between conditions for the behavioural parameters analysed (*Rproj*: *F*_3,67_=0.41, *p*=0.74; time in ROI: *F*_3,67_=0.33, *p*=0.80; speed: *F*_3,67_=0.38, *p*=0.74; figure 2*a*−*c*).

### 3.2 Bystanders' behaviour during the fights

**Figure 3.**
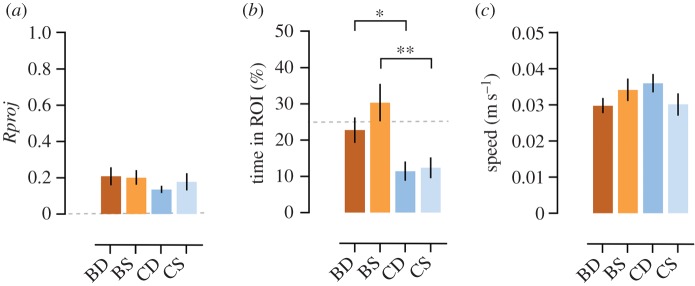
Behavioural results at the fight-observed/fight-not-observed stages. (*a*) *Rproj*in arena. Dashed grey line indicates no directionality. (*b*) Mean time spent in ROI. Dashed grey line indicates chance level (25%). (*c*) Mean speed in the arena. Conditions: BD, BS, CD and CS. Mean±s.e.m. represented. *p<0.05, **p<0.01.

During the fight interactions, directional focus towards the stimulus and mean speed in the arena were not significantly different across conditions (*Rproj*: *F*_3,67_=0.65, *p*=0.56; speed: *F*_3,67_=1.28, *p*=0.29; figure 3*a*,*c*). Control fish spent significantly less time in the ROI than bystander fish irrespective of social status (*F*_3,67_=5.92, *p*=0.001; contrasts (BD−CD): *t*_67_=2.23, *p*=0.03; *d*_s_=0.73; contrasts (BS−CS): *t*_67_=3.39, *p*=0.001; *d*_s_=1.15; figure 3*b*).

Analysis of the demonstrator dyads' latencies to fight (224.50±39.93 s, *n*=71) and fight resolution times (353.38±45.98 s, *n*=71) did not reveal any differences across conditions (latency to fight: *F*_3,67_=0.48, *p*=0.70; fight resolution: *F*_3,67_=0.61, *p*=0.60).

### 3.3 Bystanders' behaviour after the fights

**Figure 4.**
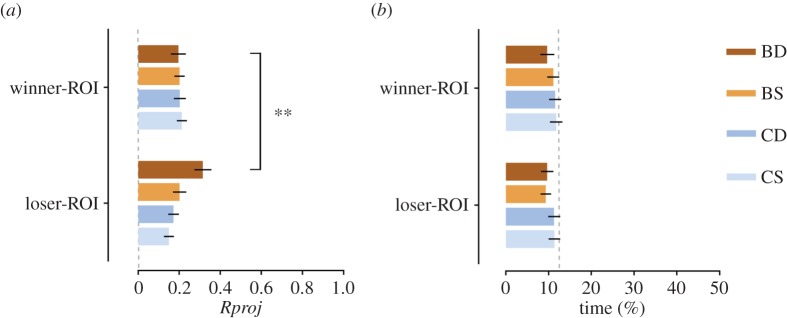
Behavioural results at the post-fight stage. (*a*) Post-fight mean directional focus (*Rproj*) towards the winner and loser demonstrator fish, in the winner-ROI and loser-ROI, respectively, for each condition. Dashed grey line indicates no directionality (zero value). (*b*) Post-fight mean time spent in the winner-ROI and loser-ROI, respectively, for each condition. Dashed grey line represents chance level (12.5%). Conditions: BD, BS, CD and CS. Mean±s.e.m. represented. **p<0.01.

In the post-fight stage, the mixed-model ANOVA revealed a main effect of treatment for the directional focus (bystanders>controls; figure 4*a* and table 1). Planned comparisons showed that dominant bystanders had a significantly higher directional focus towards losers of observed fights than towards winners (figure 4*a* and table 1). Subordinate bystanders, however, showed no differences in directional focus towards winners or losers and neither did dominant and subordinate control fish. All conditions were oriented around 180° (electronic supplementary material, table S1). There was no effect of treatment and status on the time spent in the winner and loser ROIs, with no differences detected between the two regions, for any condition (figure 4*b* and table 1).

**Table 1.** Mixed-design ANOVAs and planned comparisons of the measured behavioural parameters between the winner-ROI and loser-ROI. Bystander dominant (BD, *n*=19), bystander subordinate (BS, *n*=17), control dominant (CD, *n*=18), control subordinate (CS, *n*=17).

	winner-ROI versus loser-ROI					
	*Rproj* (−1 to 1)			time (%)		
	*F*_1,67_	*p*		*F*_1,67_	*p*	
treatment	4.67	0.03		1.39	0.24	
status	2.28	0.14		0.08	0.78	
side	0.03	0.86		1.13	0.29	
treatment×status	0.96	0.33		0.02	0.88	
treatment×side	6.39	0.01		0.23	0.63	
status×side	3.37	0.07		0.68	0.41	
treatment×status×side	0.75	0.39		0.44	0.51	
planned comparisons	*t*_67_	*p*	*d*_*z*_	*t*_67_	*p*	*d*_*z*_
BD	2.80	0.006	0.64	0.03	0.98	0.01
BS	0.00	1.0	0.00	1.48	0.14	0.36
CD	0.69	0.49	0.16	0.21	0.83	0.05
CS	1.62	0.11	0.39	0.36	0.72	0.09

Comparisons between pre-fight and post-fight (table 2) showed that observing a fight significantly increased the directional focus of BD fish towards the demonstrator fish but decreased it for BS fish (figure 2*a*). Dominant and subordinate control fish, which did not observe the fight, also showed a decrease in directional focus, although not statistically significant for the subordinates. All conditions were oriented around 180° (electronic supplementary material, table S1). No differences were detected between stages in the time spent in the ROI, for any condition (figure 2*b* and table 2). BD fish significantly decreased their mean speed in the arena in the post-fight stage, while no differences were found for the remaining conditions (figure 2*c* and table 2).

**Table 2.** Mixed-design ANOVAs and planned comparisons of the measured behavioural parameters between the pre-fight and post-fight stages. BD (*n*=19), BS (*n*=17), CD (*n*=18), CS (*n*=17).

	pre-fight versus post-fight								
	*Rproj* (−1 to 1)			time ROI (%)			speed (m s^−1^)		
	*F*_1,67_	*p*		*F*_1,67_	*p*		*F*_1,67_	*p*	
treatment	3.61	0.06		0.06	0.82		0.07	0.79	
status	0.74	0.39		0.05	0.82		1.43	0.24	
stage	2.62	0.11		0.52	0.47		2.12	0.15	
treatment×status	0.57	0.45		0.37	0.55		0.82	0.37	
treatment×stage	2.21	0.14		2.93	0.09		6.59	0.01	
status×stage	3.23	0.08		0.01	0.92		0.50	0.48	
treatment×status×stage	7.67	0.007		0.67	0.42		0.60	0.44	
planned comparisons	*t*_67_	*p*	*d*_*z*_	*t*_67_	*p*	*d*_*z*_	*t*_67_	*p*	*d*_*z*_
BD	2.30	0.02	0.53	0.14	0.88	0.03	2.85	0.006	0.65
BS	2.30	0.02	0.56	0.83	0.40	0.20	1.24	0.22	0.30
CD	2.06	0.04	0.48	0.76	0.45	0.18	0.59	0.55	0.14
CS	1.04	0.30	0.25	1.64	0.10	0.39	0.51	0.61	0.12

Correlation analysis between the bystanders' directional focus towards the losers of the observed fights and the fights' latency or resolution times revealed no significant results for BD fish (*Rproj* loser-ROI versus latency to fight: *r*_p_=−0.37, *p*=0.11; *Rproj* loser-ROI versus fight resolution: *r*_p_=−0.14, *p*=0.54; *n*=19), or BS fish (*Rproj* loser-ROI versus latency to fight: *r*_p_=0.19, *p*=0.44; *Rproj* loser-ROI versus fight resolution: *r*_p_=0.20, *p*=0.44; *n*=17).

## 4. Discussion

In this study, we demonstrate for the first time the occurrence of social eavesdropping in zebrafish and its modulation by the bystanders' social status. After observing a fight, dominant but not subordinate bystander zebrafish became more attentive towards the losers than winners of the observed fights. Moreover, control fish that could not observe the fights did not reveal any attentional preference, regardless of their dominance status. This indicates that dominant bystanders collected information about the observed fighters during the interaction and not from any post-interaction status cues, such as possible changes in coloration or body postures [6,9]. These results also imply that zebrafish are capable of ‘true’ individual recognition and attribution of social status to individual conspecifics, as found in other fish [11].

No baseline differences were found between conditions for any of the parameters analysed, showing that behaviour towards the demonstrators prior to the fight was identical and not modulated by dominance status at that stage. However, comparison between the baseline and post-fight periods confirmed that observing a fight increased the directional focus of dominant fish towards the demonstrators, while reducing their mean speed in the arena. Conversely, the directional focus of subordinate fish decreased and activity levels were not affected, similarly to control fish, suggesting a loss of interest of these fish in the demonstrators after the fight.

During the fight observation stage, no differences were found in directional focus or mean speed in the arena between conditions. Moreover, remarkably there was no increased proximity towards the demonstrators at any stage. With the exception of control fish (which avoided the opaque partition placed during the fight period), mean values remained around chance level for all conditions and stages. Thus in our study, eavesdropping was revealed by directional focus towards a conspecific rather than by proximity, a parameter which has been often used in other studies (e.g. [5]). This suggests that behavioural outputs of eavesdropping (and of social learning in general) can be subtle and potentially overlooked in many behavioural paradigms, emphasizing the importance of using novel behavioural parameters and automated tracking methods in the study of social interactions (e.g. [12]). Particularly, in our paradigm there was no possibility of territorial intrusions or interactions after the fight, and each fish controlled an adjacent territory without being able to cross it. Also, winners and losers were not aware of the bystanders' presence, thus showing no territorial or aggressive behaviours at this stage. In this context, the fact that dominant bystanders were more focused towards the losers than winners of the fights, while not preferentially approaching or avoiding either of them, may be explained as a strategy to evaluate potential territorial expansion, focused on monitoring a weaker rival while avoiding confrontation with a neighbouring dominant one [13]. Additionally, it should be expected that the quality of the fight might provide specific information to eavesdroppers and also affect their response. For instance, the latency to start a fight might be an indicator of the level of aggressive priming, and the time it takes for a winner and a loser to emerge from the fight an indirect indicator of the differences in fighting ability of the opponents. However, we found no correlation between the dominant bystanders' increased attentiveness towards the losers of the fights and the fights' latencies or fight resolution times, which entices the use of more refined individual measures of behaviour.

In our experiment we did not individually tag the demonstrators to avoid providing unintentional cues to eavesdroppers or eliciting behavioural changes during the fights. This prevented us from analysing the demonstrators' individual behaviours during the fights [9]. Nonetheless, individual fighting performance (e.g. displays, strikes, bites and chasing) and other behavioural parameters (e.g. structure of movement) have the potential to report relevant aspects of the eavesdropped information. The recent development of new video-tracking methods allowing non-invasive individual tagging of unmarked individuals [14], and the successful manipulation of video stimuli using fish [8,15], can provide the necessary tools to further develop this paradigm in future studies.

In conclusion, this study demonstrates the modulation of acquired public information by individual past social experience, which may be a fundamental process in social learning mechanisms. Given the growing number of neurogenetic tools available for zebrafish, which allow the visualization and manipulation of neural circuits in relation to behaviour (e.g. [16]), together with the development of new tracking and stimulus manipulation tools, the demonstration of social eavesdropping in zebrafish sets the stage for the study of the neural mechanisms underlying social learning in a model organism.

## Supplementary Material

Supplementary material:detailed methods section

## Supplementary Material

Eavesdropping_RSOS_Rawdata resubmission.xlsx

## Data accessibility

The behavioural datasets are available as electronic supplementary material. The custom-made scripts' source codes for positional and directional video tracking, tutorials and a test sample video are available online at https://github.com/joseaccruz/fishtracker.
