# Supplementary material for: Social dominance modulates eavesdropping in zebrafish
Source: R Soc Open Sci. 2015 Aug 26;2(8):150220. doi: 10.1098/rsos.150220 (PMC4555855; doi:10.1098/rsos.150220)
Supplement: Supplementary material:detailed methods section [file rsos150220supp1.docx]

**Social dominance modulates eavesdropping in zebrafish**

Rodrigo Abril-de-Abreu, Ana S. Cruz, Rui F. Oliveira

Supplementary Material

# Material and Methods (extended version)

**Animals and Housing**

Wild-type (AB) male zebrafish (*Danio rerio*), 9 to 12 months old, bred at Instituto Gulbenkian de Ciência (IGC, Oeiras, Portugal) were used. Fish were kept in mixed sex shoals of 30 individuals in environmentally enriched (gravel substrate, artificial plants, rocks and refuges) stock tanks with 50 × 25 × 30 cm (30 l) at 25 ^◦^C, under a 12L:12D photoperiod. Water was filtered and monitored for nitrites (< 0.2 ppm), nitrates (< 50 ppm) and ammonia (0.01 – 0.1 ppm). Fish were fed three times per day with commercial food flakes in the morning and with freshly hatched *Artemia salina* twice in the afternoon, except on the day of the experiments. All procedures were reviewed by the Instituto Gulbenkian de Ciência Ethics Committee and approved by the competent Portuguese authority (Direcção Geral de Alimentação e Veterinária permit 008955).

**Status manipulation setup**

Two fight tanks (15 × 15 cm), with a 9 cm water depth, were placed inside a bigger tank
(50 × 25 cm) containing a mixed sex shoal of 30 individuals to act as an audience (figure S1). Each fight tank was divided in half by an opaque removable partition. When lowered, the partition prevented visual and physical contact between two isolated fish but allowed chemical communication. When lifted, the fish could interact and fight. The audience allowed the fighting fish to assess their dominance status in a shoal-like context, similar to their “natural” stock tank environment, while also reducing their stress levels prior to the interaction. A camera placed in front of the setup video recorded all fights.

**
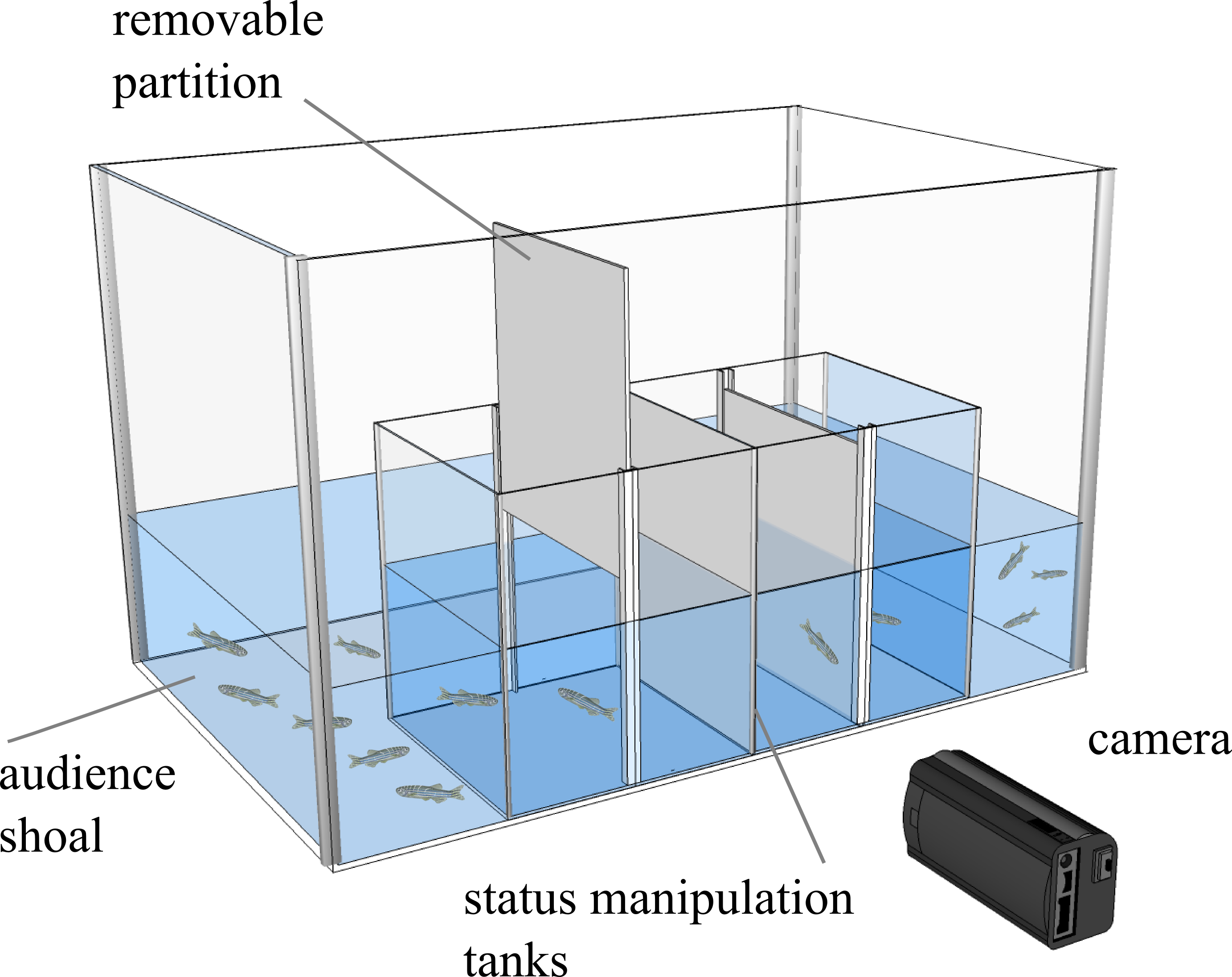
**

**Figure S1.** 3D schematic of the status manipulation setup.

**Eavesdropping setup**

A test tank (13 × 13 × 17 cm) was placed facing a demonstrator tank (30 × 15 × 17 cm),
with a one-way mirror in-between (see main text, figure 1a). This allowed a bystander focal fish placed in the test tank to see a demonstrator fish pair without itself being seen. It also prevented interactions between demonstrators and bystanders. Both tanks were filled up to a 9 cm water height. No chemical communication was possible as the tanks were self-contained. A LED light was placed over the demonstrator tank to create differential lighting required for the mirror effect. To further enhance this effect and also avoid interference of externalvisual cues, the demonstrator tank had white opaque walls and the test tank had black walls (main text, figure1a). The demonstrator tank was divided in half by a transparent partition. The outer-half (buffer tank) buffered the fish from interference of spurious external cues and minimized stress from the experimenter’s manipulations; the half adjacent to the test tank (demo tank) was further divided in two by an opaque removable partition and held the demonstrator fish. The removable partition was raised and lowered by a string-pulley system. When lowered, the partition prevented visual and physical contact between the two demonstrators but allowed chemical communication. A B&W mini CCTV camera (Henelec 300B, 420 TVL) with infrared sensitivity (IRs) was positioned above the test tank and connected to a laptop (HP Pavilion g6) to allow top-down view video recording of the focal fish. A second camera (SONY Handycam DCR-SR58E) was placed in front of the demonstrator tank (with the buffer tank in-between) and used to record the fighting interactions and post-interaction periods. The setup was placed over an infrared LED (850 nm) custom built lightbox to increase contrast between the background of the test tank and the focal fish (when video recording from above), without interfering with the fish’s vision as IR light falls outside zebrafish’s wavelength sensitivity. This optimized image quality for offline tracking of the focal fish’s behavior, using a custom made video-tracking system. The complete experimental setup comprised four adjacent replicas of the described setting, one for each experimental condition. A black curtain separated the setup from the rest of the behavioural room during the experiment.

**Experimental procedure**

On day 1 (main text, figure 1b), two pairs of unfamiliar male zebrafish matched in size were removed from their stock tanks and placed in the status manipulation setup in the two fighting tanks. Each fish from the pair was separated by an opaque partition, and allowed to habituate overnight to its half of the corresponding fight tank, with full view of the audience shoal.

On day 2, the opaque partitions were lifted so the fish dyads could fight while being recorded by the front camera. Under these experimental conditions, male zebrafish typically engage within minutes into a stereotypical structured fight for dominance (see supplementary movie 1), which results in a clear winner and loser of the fight. Once the fight was resolved and winners and losers emerged, they were again separated by the opaque partition.
The video recordings were analyzed to identify the acquired dominance status (dominant
or subordinate) of each fish. They were distinguishable at this point, since the winners (labeled dominants) exhibit aggressive behaviours like chasing, biting and striking, whereas the losers (labeled subordinates) flee and display submission and freezing postures. Two dominants and two subordinates were obtained from these two interactions to be used as focal fish. They were then individually placed in the test tanks of the eavesdropping setup and randomly assigned to bystander or control treatments. Therefore, four focal conditions were created: bystander dominant (BD), bystander subordinate (BS), control dominant (CD), control subordinate (CS). In parallel, four male pairs matched in size, were removed from their stock tanks and placed in pairs in each demonstrator tank to be used as fighters, separated by an opaque partition. Each focal fish could see the corresponding demonstrator pair through the one-way mirror to allow familiarization. All fish were left to habituate overnight.

On day 3, the eavesdropping test (main text, figure 1b,c) started with a 30 minutes pre-fight stage (baseline), where each focal fish had full view of the separated demonstrators. It was followed by a 30 minutes fight-observed stage for the bystander treatment fish and
a fight-not-observed stage for the control fish. Here, bystanders were allowed to observe
a fight interaction between the respective demonstrator pair while controls were prevented from it by an opaque partition blocking the view. Afterwards, winners and losers were again separated by the opaque partition. The fights were video recorded with the front camera,
for later determination of the winner and loser’s random end position in their tank (left
or right), after the lowering of the partition. In the final post-fight stage, the partitions that blocked the view of the control fish were removed and all focal fish were allowed to observe for 30 minutes the winners and losers of the corresponding fights. During this time period
no interaction occurred between the winners and losers, as they remained separated by
an opaque partition. Focal fish were video recorded at all stages. On rare occasions demonstrator fish did not resolve the fight or the video recordings malfunctioned. In such cases the corresponding focal fish were discarded prior to analysis. A total of 71 focal fish were analyzed (n = 19 for the BD condition; n = 17 for BS; n = 18 for CD; and n = 17 for CS).

**Behavioural tracking and data acquisition**

All focal fish were tracked (see supplementary movie 2) at the pre-fight and post-fight stages from a top-down view, using a custom made tracking software developed in Python (python^TM^). For each behavioural video, a 2D region (arena) was defined for tracking
(main text, figure 1c). The arena’s position and size took into account the perspective distortion caused by water depth. It comprised the inner area of the test tank (12 × 12 cm). Each fish was video recorded and tracked at a 25 fps rate. For each frame, the tracking software determined and extracted into data files the pixel coordinates of the head, centroid, and tail of the fish. This allowed determination of the position and orientation of the fish every 1/25 s.

**Behavioural Analysis**

All tracked data files were imported to MATLAB (MathWorks) and behavioural parameters were determined using a custom-made script. Eavesdropping effects were investigated at the post-fight stage by comparing behaviour in two defined regions of interest closest to the winner (winner-ROI) and loser (loser-ROI) demonstrator’s sides (figure 1c). Each region had 6 × 3 cm, corresponding to 12.5% of the tank. Directional focus towards
the demonstrator (*Rproj*), time spent in each region and mean orientation angle (), were determined for each focal fish and condition. *Rproj* was defined as the projection of the fish’s mean resultant directional vector’s length *R* (figure 1d) onto the demonstrator tank’s direction (180^o^). The mean resultant vector was calculated by first transforming each direction taken by the fish during the 30 minutes test into a unit vector , where is the angle formed by the fish’s centroid-to-head axis relative to the horizontal axis in each frame. The mean resultant vector was thus defined as the mean of all *n* frames unit vectors, calculated by , and the corresponding directional focus was measured by the mean resultant vector’s length , which is defined by the vector’s norm and inversely related to the angular standard deviation. Its values range from 0 to 1. The closer is to one, the more focused are the *n* orientations around the mean direction. Finally, *Rproj* was determined by , where *α* is the mean resultant vector’s angle (main text, figure 1d) relative to the horizontal axis. This allowed measurement of the mean directional focus of each fish relative to the stimulus direction, using a linear scale ranging from 1 to -1. Positive values indicate directionality towards the stimulus direction, negative values away from it and null values no directional focus.

Mixed-design ANOVAs were used to check effects and interactions of treatment, status and side (within-subjects variable) on the attentional measures. Planned contrasts were used within the model for specific comparisons between the winner-ROI and loser-ROI. Effect sizes were determined by Cohen’s d.

Baseline (pre-fight) values of *Rproj* and mean speed (measure of motor activity) were determined in the total arena (tracked area) together with the time spent in a region of interest (ROI) closest to the demonstrator tank (main text, figure 1c). The ROI had 12 × 3 cm (25 % of the tank), corresponding to the width of the arena and the mean body length of an adult zebrafish.
One-way ANOVAs were used to compare all conditions. Trend effects from observing
or not observing a fight, were analysed by comparing pre-fight with post-fight for each condition. Mixed-design ANOVAs and planned contrasts were used.

The demonstrator fishes’ latency to fight (time to first aggressive display) and fight resolution time (from first display to winner-loser decision) were determined for all dyads, and the focal fishes’ behaviour was analysed during the fights. One-way ANOVAs were used to compare all conditions. Pearson correlations were performed between the latency to fight, resolution times, and subsequent levels of bystander fishes’ directional focus towards the losers at the post-fight stage.

Behavioural parameters were represented as mean ± SEM, except mean angles represented as mean ± 95% C.I., when directionality was significant. Statistical significance was considered for *p* < .05. All analyses were performed using MATLAB R2012b (MathWorks) with the CircStat toolbox, STATISTICA 12 (Statsoft, Inc.), SPSS Statistics 22 (IBM), and Oriana 4 (Kovach Computing Services).

# Supplementary Table S1

**Table S1.** Mean orientation angles (mean, 95% CI).

|  | **winner-ROI** | **loser-ROI** | **pre-fight** | **post-fight** |
| --- | --- | --- | --- | --- |
|  | **α (^o^)** | | | |
| BD | 175.87, [130.07, 233.63] | 192.16, [161.04, 216.87] | 184.67, [173.14, 194.40] | 182.73, [170.65, 206.22] |
| BS | 165.65, [125.77, 213.65] | 193.71, [137.42, 223.93] | 171.81, [161.29, 189.85] | 189.07, [158.89, 212.40] |
| CD | 199.17, [148.26, 231.22] | 187.11, [142.07, 238.55] | 172.39, [157.01, 227.34] | 177.98, [154.28, 226.82] |
| CS | 207.73, [163.05, 235.95] | 191.65, [127.63, 242.18] | 172.05, [156.01, 247.63] | 183.35, [162.23, 230.32] |
| BD – bystander dominant (n=19), BS – bystander subordinate (n=17), CD – control dominant (n=18), CS – control subordinate (n=17). | | | | |
